# Dynamic Tendon Grip (DTG™) novel knot array compared to traditional sutures for zone two flexor tendon injury – a biomechanical feasibility study

**DOI:** 10.1186/s12891-022-05279-9

**Published:** 2022-04-04

**Authors:** Assaf Kadar, Alon Fainzack, Mordechai Vigler

**Affiliations:** 1 Rabin Medical Center, 39 Jabotinski, Petach Tikva, Israel; 2 Affiliated with the Sackler Faculty of Medicine, Tel Aviv University, 49100 Tel Aviv, Israel

**Keywords:** Biomechanical study, Early active motion, Dynamic tendon grip device, Flexor tendon, Tendon injuries

## Abstract

**Background:**

Flexor tendon injuries pose many challenges for the treating surgeon, the principal of which is creating a strong enough repair to allow early active motion, preserving a low-profile of the repair to prevent buckling and subsequent pulley venting. A main concern is that a low-profile repair is prone to gap formation and repair failure. The Dynamic Tendon Grip (DTG™) all suture staple device claims to allow a strong and low-profile repair of the flexor tendon. The purpose of this study is to test the effects of the DTG™ device in early active motion simulation on range of motion, load to failure and gap formation and to compare it to traditional suturing technique.

**Methods:**

Twelve fresh-frozen cadaveric fingers were assigned to two groups: DTG™ device (*n* = 9) and traditional suturing (double Kessler 4-core suture and a peripheral suture, *n* = 3). The deep flexor was incised and repaired in zone 2, and active motion simulation was carried out with a cyclic flexion–extension machine. Finger range of motion and gap formation were measured, as well as load to failure and method of repair failure.

**Results:**

Following motion simulation, ROM decreased from 244.0 ± 9.9° to 234.5 ± 5.8° for the DTG™ device compared to 234.67 ± 6.51° to 211.67 ± 10.50° for traditional suturing. The DTG™ repair demonstrated gap formation of 0.93 ± 0.18 mm in 3 of 8 specimens after applying 1 kg load, which negated after load removal. Load to failure averaged 76.51 ± 23.15 N for DTG™ and 66.31 ± 40.22 N for the traditional repair. Repair failure occurred as the suture material broke for the DTG™ array and at the knot level for the traditional repair.

**Conclusions:**

The DTG™ all-suture stapling concept achieved a strong low-profile repair in zone 2 flexor tendon injury after active motion simulation. Further clinical studies will be needed to determine the effectiveness of this device compared to traditional techniques.

**Supplementary Information:**

The online version contains supplementary material available at 10.1186/s12891-022-05279-9.

## Introduction

Flexor tendon injuries of the hand, especially in zone 2, account for less than 1% of all hand injuries, but are difficult to treat and are associated with poor outcome [1]. While major progress has been made with the treatment of these injuries, current surgical treatment relies mostly on conventional suturing techniques with dissatisfactory results, concluding in re-operation rates of 6%-17%, and complication rate of up to 20% [1–5]. Furthermore, a considerable wide diversity in suturing techniques used by different health providers, might imply a superior technique has yet to be discovered [1, 4, 6, 7].

The principal challenge for the treating surgeon is creating a strong enough repair to allow for early active motion. Preserving a low-profile repair to prevent tendon bulge and subsequent pulley venting is another challenge as low-profile repair is prone to gap formation and repair failure [8, 9]. New techniques and devices have been proposed and tested to address the challenges of zone 2 flexor tendon injuries, among them are the TenoFix™ anchor-coil system, that is currently off the market, and the recent FDA approved CoNextions® TR Tendon Repair System, both are based on metallic anchors.

**Fig. 1:**
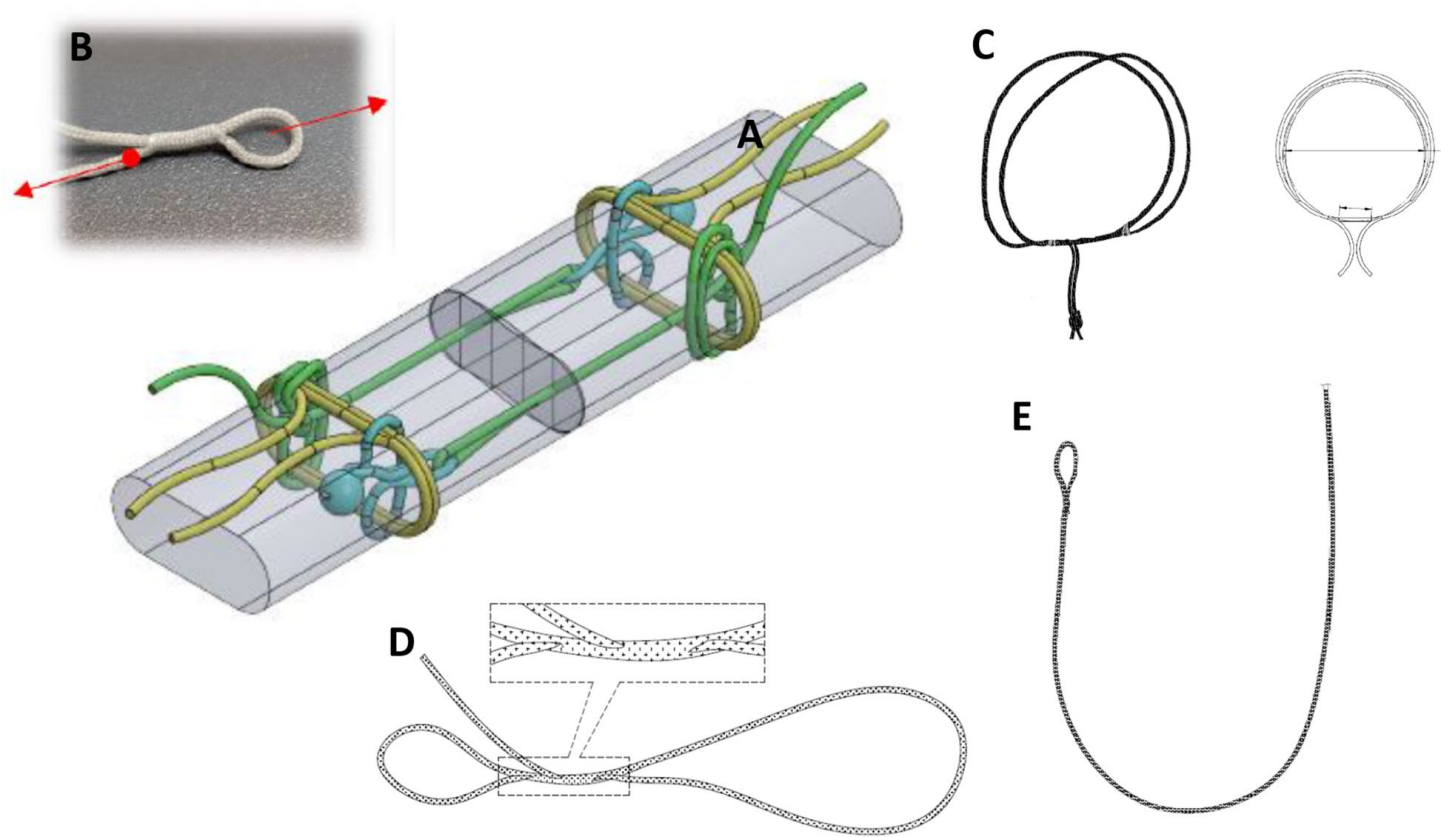
The Dynamic Tendon Grip suture array (**A**) is based on several Whoopie Sling (**B**). WS is an adjustable loop that locks once under stress, therefore functions as a ratchet. Once its size is set and traction is applied (see RED arrows), the sling is tightened and locks the knot. Bracing Double Ring [DBR] (Yellow Element): The ring establishes the grip of the implant in the tendon (**C**). Whoopie Sling with Brummel Eye (Green Element): The fixation array uses two WS components. Their function is to allow approximation and alignment between the two parts of the tendon (**D**). Soft Shackle [SS] (Cyan Element): the SS connects between the WS and the BDR (**E**)

The Dynamic Tendon Grip (DTG™) is an all-suture tendon stapling device. The device is based on a whoopie sling (WS) that comprise adjustable “Bracing Double Ring” and two adjustable “Separate loops”. This device allows a circumferential clutch of the tendon stump by the “Bracing Double Ring” and controlled approximation and alignment between the two stumps by the two “Separate Loops” (Fig. 1). This novel knot array, that will be applied with a dedicated applicator, claims to preserve the tendon profile and allow a low-profile, robust flexor tendon repair that will be faster and more reproducible than the traditional suture repair.

This biomechanical study’s aim was to test the feasibility of the DTG™ knot array. We compared repair strength, finger range of motion (ROM) and gap formation after application of the DTG device compared to traditional suturing of flexor tendons.

## Materials and methods

Four fresh frozen cadaveric upper extremity cut below the elbow were obtained. All cadavers were male, age averaged at 64 and BMI at 26.3. The ring, long and index finger were allocated into two groups: DTG™ device (*n* = 9) and the traditional double Kessler and peripheral suture repair (*n* = 3). In accordance with the Local Ministry of Health regulations, cadaveric studies are exempted and do not require institutional review board approval. Cadavers were supplied by Science Care (Phoenix, AZ).

**Fig. 2:**
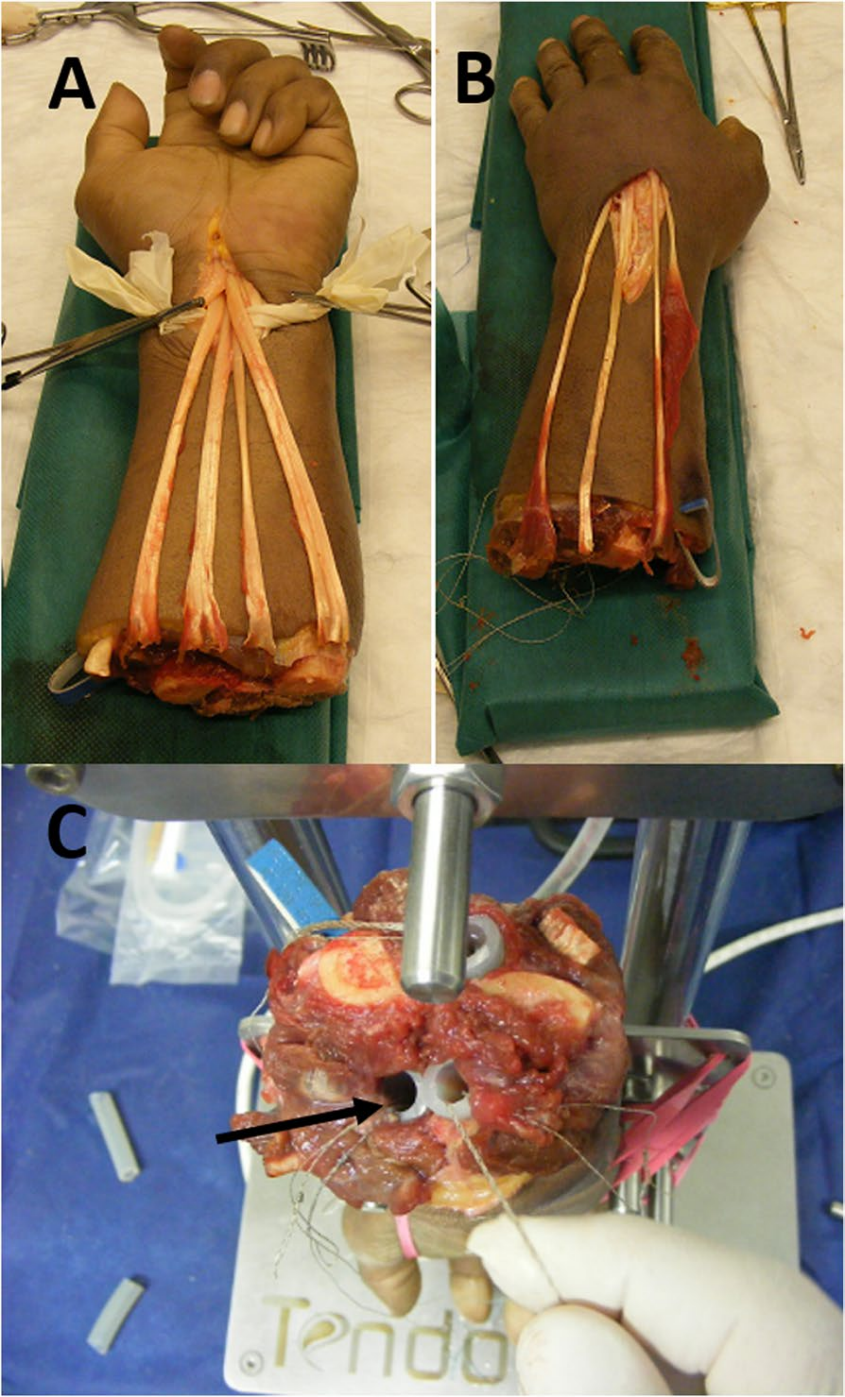
Preparation of the specimen for testing. Identifying and isolating the deep flexor tendons just proximal to the carpal tunnel (**A**); and the extensor just distal to the extensor retinaculum (**B**); all tendons were sutured proximally with a Krakow suture and were passed through silicon tubes (black arrow) to facilitated smooth motion. A saline solution was applied via the tubes to maintain tissue hydration and prevent tissue desiccation (**C**)

The deep Flexors and extensors tendons were identified and isolated at the mid forearm level to allow independent movement of each finger. The tendons were each placed in a silicon tube in order to negate friction with the surrounding tissue. The tendons were hydrated throughout the experiment with normal saline dripped to the silicon tubes to prevent tissue desiccation (Fig. 2).

A goniometer was used to measure the baseline maximal flexion angle of each joint (metacarpophalangeal joint, proximal interphalangeal joint and distal interphalangeal joint) with the maximal pull of the deep flexors.

### Surgical technique

**Fig. 3:**
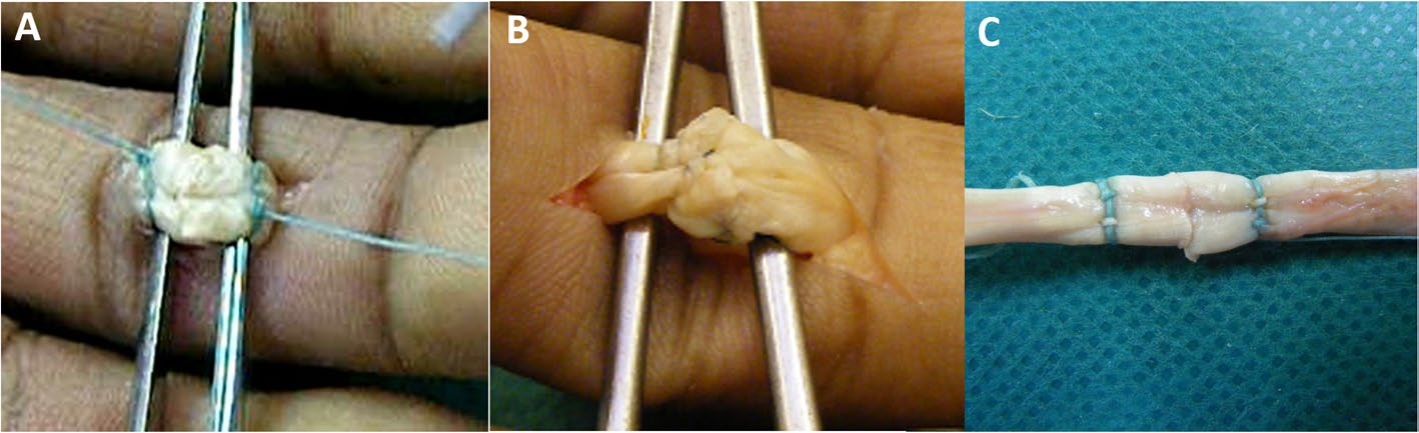
Flexor tendon zone 2 repair using the Dynamic Tendon Grip suture array (**A**); and traditional 4 strand core suture double Kessler array with 3–0 FiberWire® (2 core sutures) and 3–0 PROLENE® (2 core sutures) and a peripheral running 6–0 PROLENE® suture (**B**). Notice the typical bulging of the traditional technique compared to the low-profile of the DTG™ array (**C**)

A longitudinal incision was made over the middle phalanx. A3 pulley was incised and the flexor digitorum profundus was isolated and cut just distal to Camper’s chiasm in zone 2. Tendon cross section at zone 2 is roughly 15mm^2^ [10]. The traditional repair was performed with a 4-core strand double Kessler locking suture using a 3–0 FiberWire® (Arthrex, Naples, FL) and 3–0 PROLENE® suture (Ethicon, Inc., Somerville, NJ). A simple running peripheral suture was applied with the dorsal wall sutured first with 5–0 PROLENE® (Fig. 3).

The DTG™ repair was performed using the DTG™ knot array. The bracing double ring was applied firmly on the tendon 7 mm from the tendon edge. The next two components (soft shackle and the whoopie sling with Brummel Eye) were inserted into the tendon with a specialized needle. A knot was made in the whoopie sling with Brummel Eye, and it was threaded through the other stump and attached to the other soft shackle with another knot. This process was performed on both sides of the tendon. Finally, the whoopie Sling element was gently pulled to allow for approximation of the tendon edges (Fig. 3, Video 1). All implanted elements were comprised of a custom braided 16-strands suture: 10 strands made of Ultra-high molecular weight polyethylene (UHMWPE) and 6 strands made of polyester (Fig. 1, see supplementary material).

Pulley venting was performed based on surgeon’s discretion in order to achieve free tendon gliding. Skin was then closed with 4–0 Nylon Suture. All repairs were performed by a fellowship trained hand surgeon.

### Biomechanical study

A biomechanical study was designed to measure combined finger ROM, gap formation and repair load to failure after simulation of active rehabilitation protocol.

**Fig. 4:**
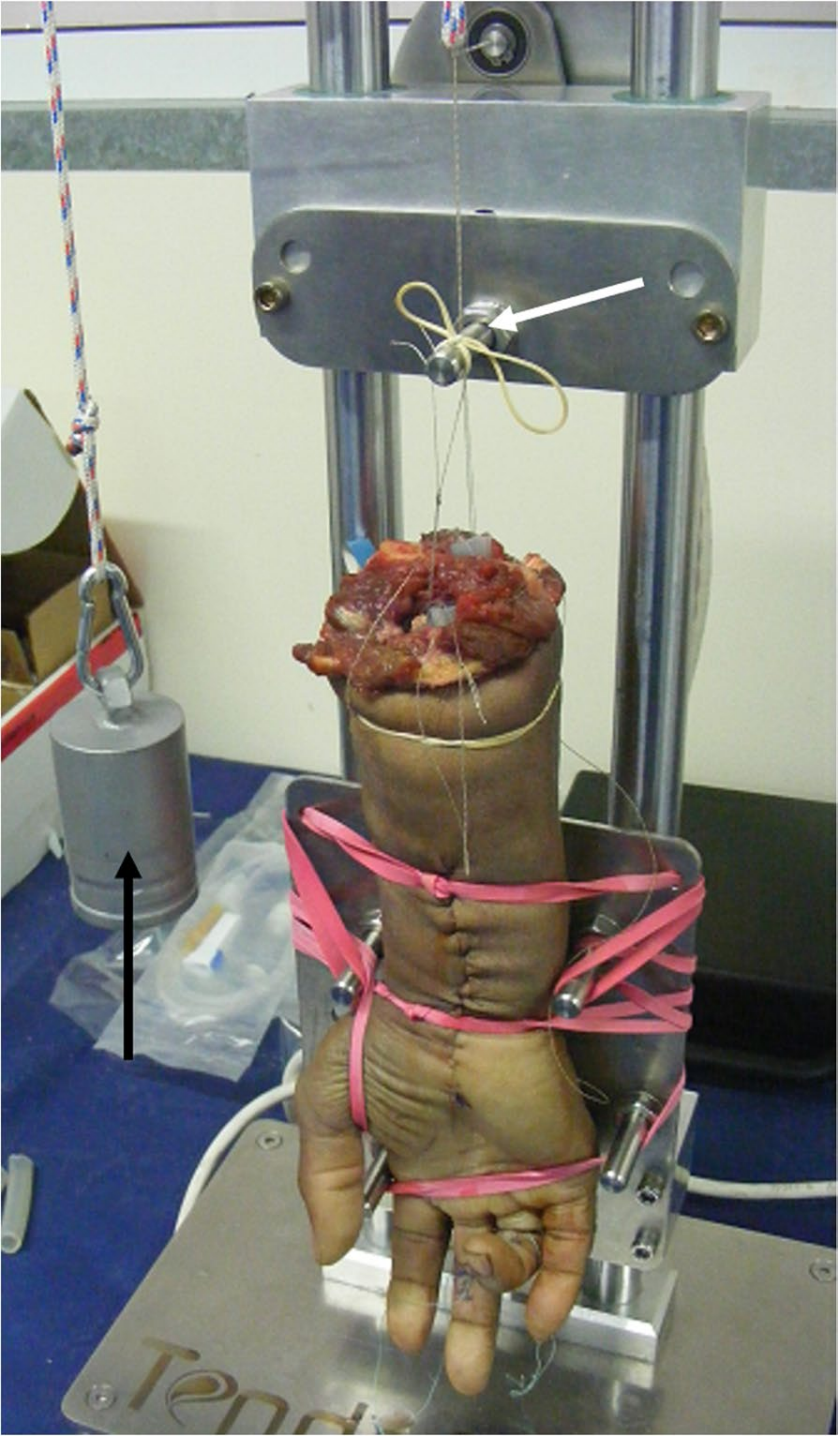
Cyclic flexion–extension using the finger motion simulator. The hand is fixed to the device. The flexor tendons are tied to the motor that provides 2 cm tendon excursion (white arrow). The extensors are tied through a pulley to a 1 kg weight (black arrow) that allows straightening of the finger when the flexor load is removed

The hand was mounted and fixed to a cyclic flexion–extension machine capable of producing 2 cm of motion. The deep flexors were attached to an actuator in order to produce full flexion, and the extensors tendons were attached to a 1 kg weight through a pulley to allow for static load and finger extension. The use of 1 kg weight to provide full finger extension has been used in our study similarly to other studies [11]. A visual confirmation of full extension and flexion was obtained for each cycle. Of note, some authors have recently claimed that a force of 2 kg is needed to simulate finger motion, however, we did not find it necessary for our study [12]. Each repair was cycled for 2,000 cycles of flexion–extension at a rate of 0.3 Hz, simulating active rehabilitation motion protocol [13] (Fig. 4).

Following the simulation, combined ROM (i.e., the MCP, PIP and DIP joints) with pull of the deep flexors was measured with a goniometer and compared to the pre-operative range. Next, the incision was opened, and gap formation of the repair site was measured with an electronic caliper with 1 kg static load applied. If gap was present with a 1 kg load, the load was then removed, and closure of the gap was assessed. Finally, the tendon was removed from the hand and load to failure was assessed with an electronic dynamometer. Additionally, the method of failure (i.e., at the knot, at the suture or at the tendon) was documented.

#### Statistical analysis

 Mean and standard deviations were used for descriptive statistic. Continuous variables were compared using the Student t-test (unpaired) understanding its limitations due to the small sample size. Level of significance was set at < 0.05.

## Results

Combined finger ROM after simulation of active rehabilitation protocol for the traditional suture group decreased significantly from 234.67 ± 6.51° to 211.67 ± 10.50° (*p* = 0.04) compared to a non-significant decrease of 244.0 ± 9.9° to 234.5 ± 5.8° (*p* = 0.20) for the DTG™ device (Table 1). Distal A2 pulley venting was required for all traditional repairs due to the bulge of the repair preventing smooth gliding under the pulley. No venting was needed for the DTG™ group, due to visible low-profile of the repair.

**Table 1:** Biomechanical comparison of range of motion, gap formation and load to failure between DTG array and traditional flexor tendon suture

	**Combined Range of motion (deg)**		**Gap formation (mm)**	**Load to Failure (N)**
	Pre-operative	Post-operative		
**DTG suture array**^**a**^	244.0 ± 9.9	235.62 ± 9.4	0.31 ± 0.48	76.51 ± 23.15
**Traditional flexor tendon suture**^**b**^	234.67 ± 6.51	211.67 ± 10.50	0	66.31 ± 40.22

^**a**^Dynamic Tendon Grip

^**b**^Traditional repair was performed with a 4-core suture Kessler array, with 3–0 FiberWire® (2-core sutures) and 3–0 PROLENE™ (2-core sutures) and a peripheral running 6–0 PROLENE™ suture

Gap formation under static 1 kg load was 0.31 ± 0.48 mm for the DTG™ device and occurred in 3 specimens. There was no gap for the traditional suture group (*p* = 0.30). Gap formation for the DTG™ group was a dynamic phenomenon, and the gap re-coiled after load removal.

**Fig. 5:**
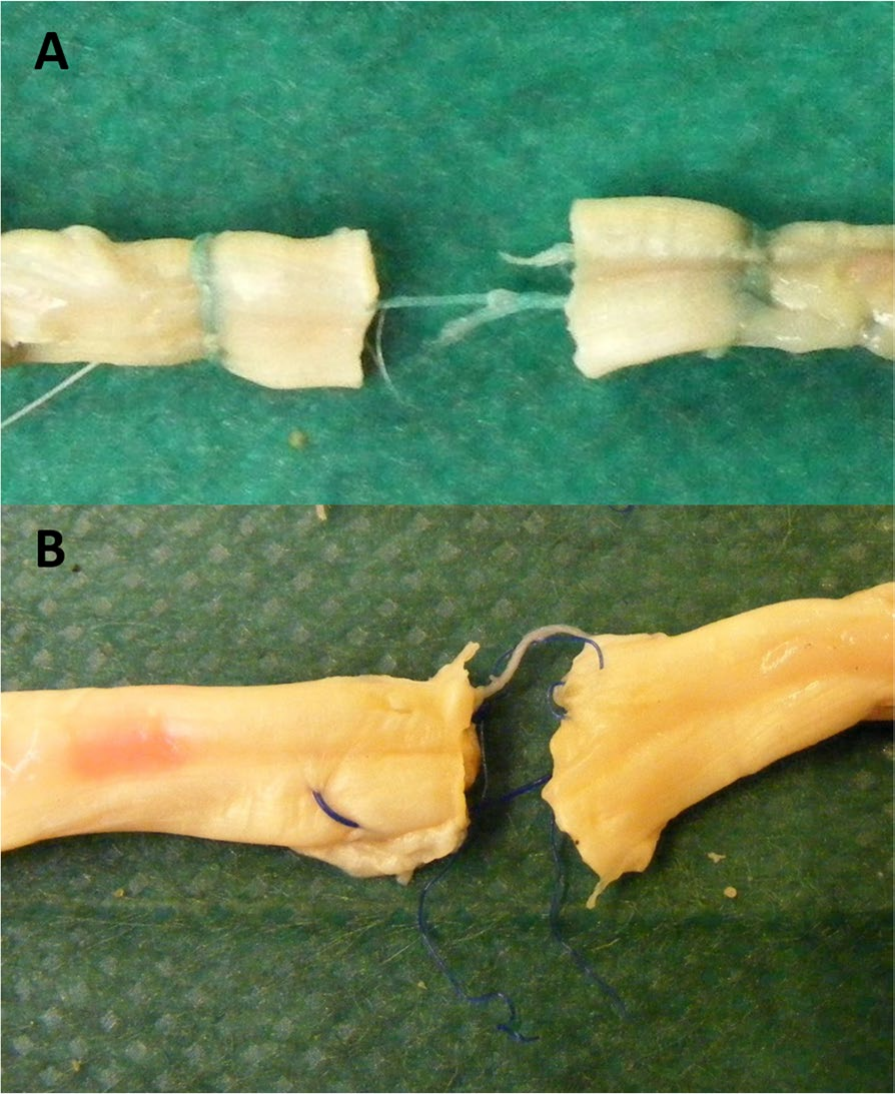
Method of repair failure was by suture failure for the DTG™ device (**A**) and by knot failure for the traditional four strand repair (**B**)

Load to failure of the traditional suture was 66.31 ± 40.22 N compared to 76.51 ± 23.15 N for the DTG™ device (*p* = 0.61). Repair failure occurred at the knot for all traditional repair, and at the suture material for all DTG™ repair (Fig. 5).

## Discussion

The Dynamic Tendon Grip (DTG™) is a novel all suture flexor tendon stapling device. In this biomechanical feasibility study, we found that the DTG™ device withstood simulation of an active rehabilitation protocol with several advantages over the traditional suture technique. The low-profile repair allowed for better combined range of motion and for pulleys to be kept intact. Load to failure was similar for the two groups. Moreover, the fact that failure occurred at the suture and not the knot makes this technique less dependent on surgeon’s skill and more dependent on stronger suture materials. The DTG™ repair was prone for gap formation at the repair site, although the gap formed was less than 1 mm.

Pulley venting is controversial in the hand surgery literature. Some surgeons meticulously preserve the pulleys to prevent bowstringing, whilst other surgeons vent both A4 and a large part of A2 [14]. Biomechanical studies have consistently demonstrated that venting of the pulleys increase work of flexion due to bowstringing [11, 15]. Yet, clinical practice exhibits no adverse effects of pulley venting [7, 14, 16, 17]. One of the reasons for pulley venting is that a low-profile repair, not requiring venting, is prone to gap formation and is weaker than a bulging repair that requires venting. Some surgeons support increasing the diameter of the junction site of the two tendon ends by 20–30% [18]. We found that the DTG™ device might allow a low-profile repair that is strong enough to allow early motion without venting.

Boyer et al. [9] showed that gap formation is deleterious for flexor tendon healing. Tendon repairs with gaps of less than 3 mm accrued strength during tendon healing, whilst gaps over 3 mm showed no increase in tendon strength. The DTG™ repair was prone to gap formation which occurred in 3 of the specimens. The gap was a dynamic phenomenon and the tendon recoiled after the load was removed. We hypothesize that this phenomenon is caused by the elasticity properties of the suture material. Furthermore, as the gap size was less than 1 mm, the clinical significance of this finding is unclear. Further animal studies will be required to show if this phenomenon has an effect on the strength of the repair.

The technique used for the control group, 4- strand core suture with a epitendinous peripheral suture, has been debated to be the gold standard that allow optimal balance between repair strength, length of procedure, and also allows for quick rehabilitation [3, 5, 7, 19]. With the traditional repair, rupture occurred at the knot level, compatible with prior evidence[3, 6]. Contrarily, the DTG™ repair failed at the suture material. Authors feel this finding might hold further potential of improvements in tensile strength of the device with the advent of newer and stronger suture materials. Our finding demonstrated a repair strength of 76.51 N with the DTG™ repair, which should allow for early active motion rehabilitation protocols [20].

The need for a stronger, more reliant method, has propelled several innovations in recent years. The first FDA approved anchoring system for soft tissues was the “TenoFix™” system: A stapling system attached to each tendon stump with an anchor-coil complex, joined by a 2–0 multifilament stainless steel suture. While being regarded as relatively strong, safe and a possible alternative for noncompliant patients, its use has not become widespread primarily due to its cost, complexity of the technique, large surgical exposure and failure to sufficiently mitigate incidence of tendon rupture [7, 21, 22].

A new FDA approved tendon coupler device, CoNextions® TR Tendon Repair System (CoNextions® Medical), is made of Nitinol and UHMWPE. It was recently tested with cadaver hands and compared to an 8-strand locking-cruciate technique in repair of Zone 2 injuries [23]. The study showed similar gap formation after simulated active rehabilitation protocol, superior repair speed (1:4 ratio), and higher residual load to failure as compared to the traditional technique used. Recent clinical trial of the CoNextions® TR device demonstrated the device was at least as safe and effective as suture for the repair of lacerated Zone 2 FDP tendons [24].

Our study’s major limitation is sample size, and thus should be considered a feasibility study. Other limitations are size difference between the DTG group and control group and the pooling together of data from three different fingers, the index, middle and ring finger. As this was a feasibility study where hand surgeons attempted the DTG device for the first time to test its strengths and weaknesses, we had more specimens in the DTG group than the control group. Venting was at the surgeons’ discretion which might be biased. However, venting of the pulley to allow a bulging repair to glide smoothly is a common practice for surgeons worldwide [25]. The surgeons at this study felt that the DTG™ repair glided smoothly enough not to require any venting. Another limitation was gap formation assessment with 1 kg of load, which might be lower than what the tendon will experience in reality. Finally, cadaveric studies are inherently restricted by their inability to reproduce biological processes of healing. However, a recently performed in vivo animal experiment (unpublished data) proves that the healing process is biologically and histologically similar between the DTG device and the gold standard 4 strands suture after 3 weeks of simulated flexor tendon laceration and repair.

*In conclusion,* Within the confines of a small sample, our feasibility study showed that the DTG™ all-suture stapling concept achieved a strong low-profile repair in zone 2 flexor tendon injury. Reduced post-operative impairment of range of motion and similar load to failure compared to the gold standard four core strand repair were measured, yet, larger gap was also measured. Further animal and clinical studies will be needed to determine the effectiveness of this device compared to traditional techniques*.*

## Supplementary Information

**Additional file 1.**

**Additional file 2.**

## Data Availability

All data generated or analysed during this study are included in this published article and its supplementary files.
