# Supplementary material for: Dynamic Tendon Grip (DTG™) novel knot array compared to traditional sutures for zone two flexor tendon injury – a biomechanical feasibility study
Source: BMC Musculoskelet Disord. 2022 Apr 4;23:320. doi: 10.1186/s12891-022-05279-9 (PMC8978384; doi:10.1186/s12891-022-05279-9)
Supplement: Supplementary file 2 — Additional file 2. [file 12891_2022_5279_MOESM2_ESM.pdf]

GENERAL

The DTG is an implantable "Knots Array" comprises of an adjustable "**Bracing Double Ring**"," **Whoopie Sling with a Brummel Eye**" and a "**Soft Shackle**".

The DTG aims, on one hand, to embrace and attach the torn tendon stump (by the "**Bracing Double Ring**" and the "**Soft Shackles**") and on the other hand, to allow performing a controlled approximation & alignment between the two torn stumps (by the two "**Whoopie sling with Brummel eye**").

The **Whoopie Sling** is an adjustable loop that locks once under stress, therefore functions as a ratchet. Once its size is set and traction is applied (see RED arrows), the sling is tightened and locks the knot.

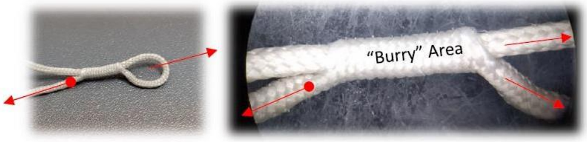

<u>Final deployment state</u> – General View

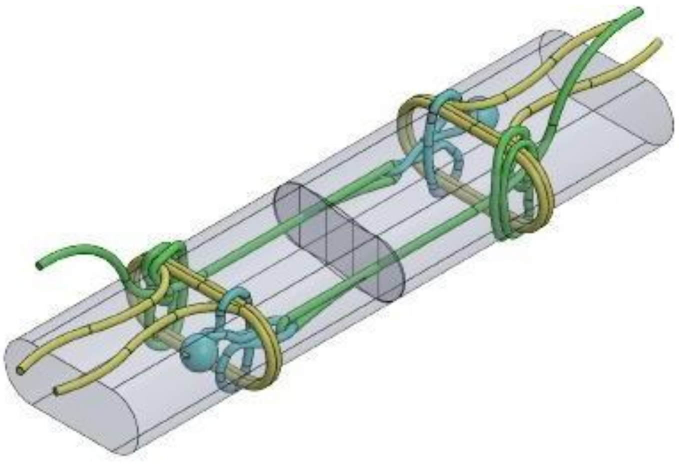

The Device Elements:

Bracing Double Ring [**DBR**] (Yellow Element): The ring establishes the grip of the implant in the tendon

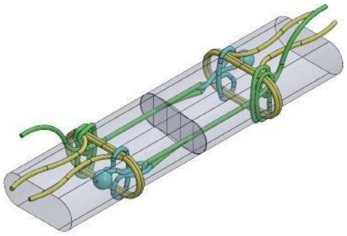

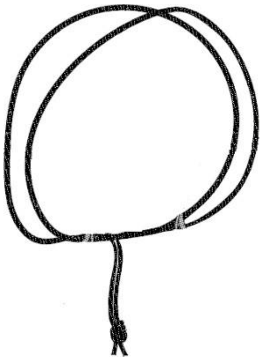

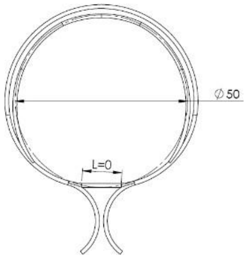

Whoopie Sling [**WS**] with Brummel Eye (Green Element): The fixation array uses two WS components. Their function is to allow approximation and alignment between the two parts of the tendon

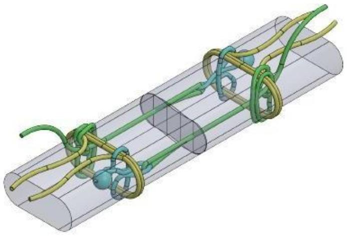

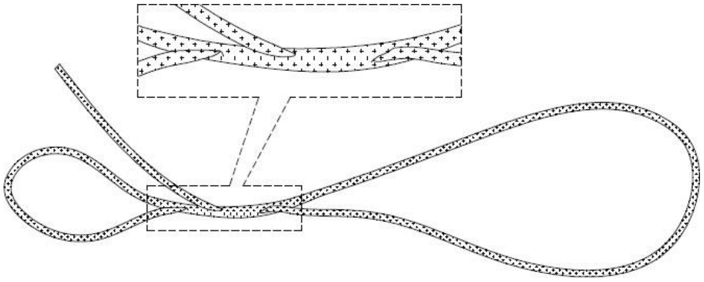

Soft Shackle [**SS**] (Cyan Element)**:** the SS connects between the WS and the BDR

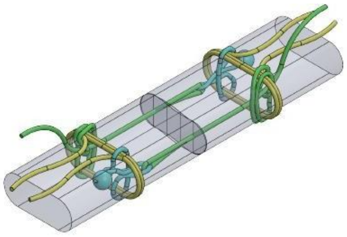

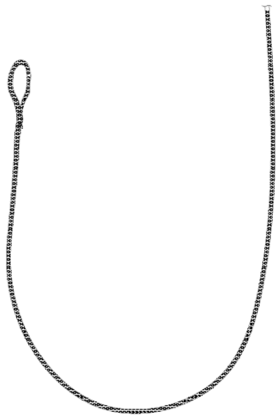

FIG. 21

Material:
All device's element (DBR, WS & SS) are made of a customed suture, braided of 16 strands of which 10 are UHMWPE and 6 are Polyester. Both materials are known and well used in the medical arena as raw material for surgery sutures.

Method of use:
The device will be deployed by the surgeon, using standard surgery suturing tools.

Figure 4A illustrates anchoring of device 10 (**DBR**) to a severed end of a tendon (T). Adjustable self-locking loops 20 and 24 of device 10 are positioned around the stump of the tendon and are tightened while maintaining the relatively oval profile (cross-section) of the tendon.

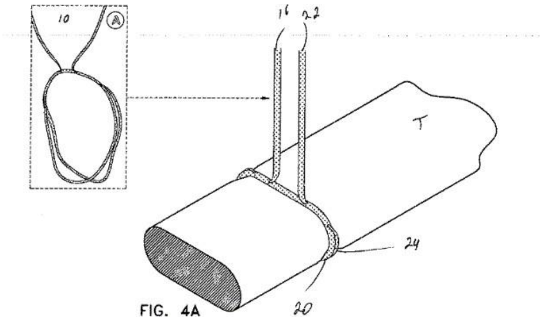

Figure 4B illustrates anchoring of device 100 (**SS**) to the tendon. Using a needle (not shown), end 102 of connector 100 is threaded into and out of the tendon around loops 20 and 24.

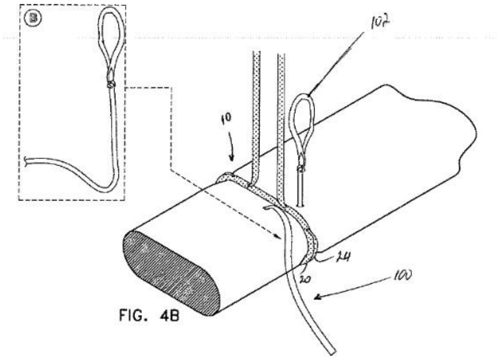

Figure 4C illustrates anchoring of tensioner 50 (**WS**) to the tendon. Using a needle (not shown), fixed loop 60 is threaded into and out of the Tendon around loops 20 and 24.

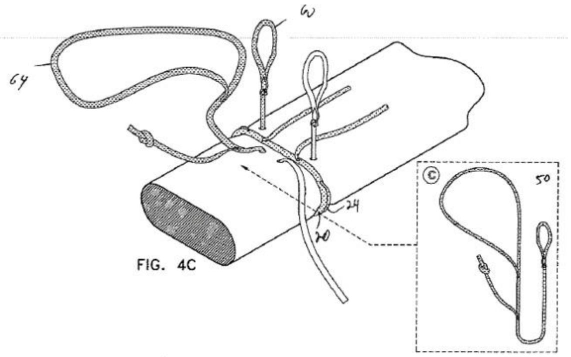

In Figures 4D-F, adjustable self-locking loop 64 of tensioner 50 is threaded inside fixed loop 60 and then into the tendon and out of the severed end using needle 65.

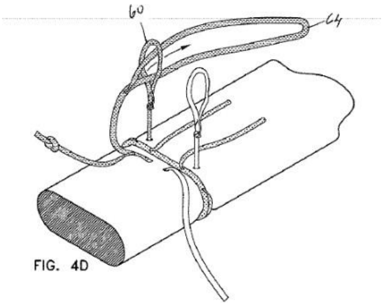

FIG. 4D

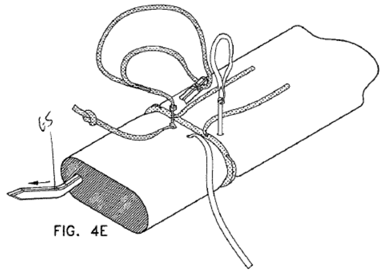

FIG. 4E

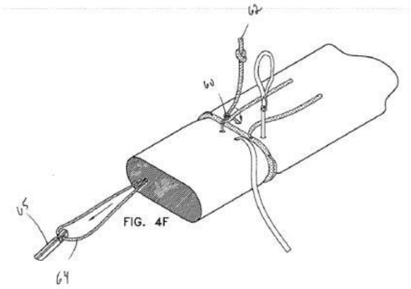

FIG. 4F

Following performing the above steps on the second severed end of the tendon, two needles 66 are inserted into and along the tendon to grip adjustable self-locking loop 64 of tensioner 50 and to pull it inside and over device 10 (Figures 4G-H).

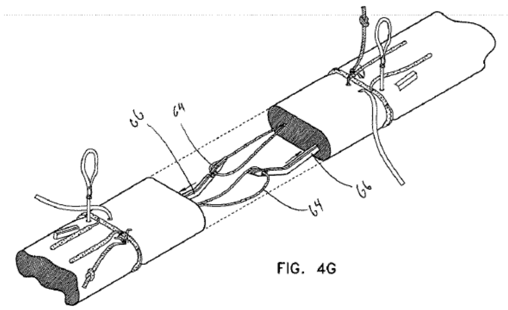

FIG. 4G

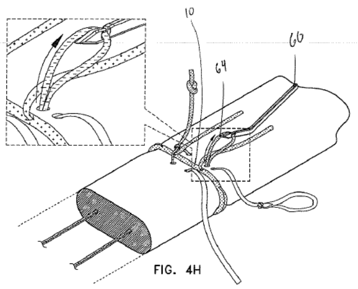

FIG. 4H

Free end 103 of device 100 is inserted through adjustable self-locking loop 64 of tensioner 50 and through fixed loop 102 of device 100 as is shown in Figures 4I-J. Excess thread of connector 100 is collected and fixed loop 102 is pooled towards the tendon surface (Figure 4K).

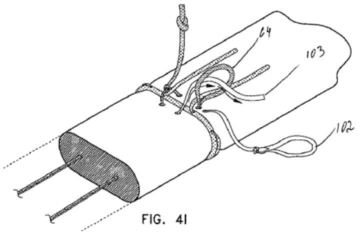

FIG. 4I

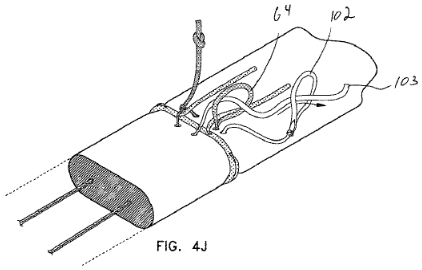

FIG. 4J

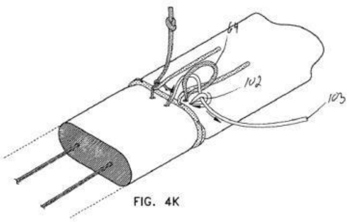

FIG. 4K

Connector 100 is then secured by performing two adjustable overhand knots. The first knot is a regular knot (Figures 4L-M) while the second knot is performed by threading free end 103 through hole 104 (Figures 4N-P).

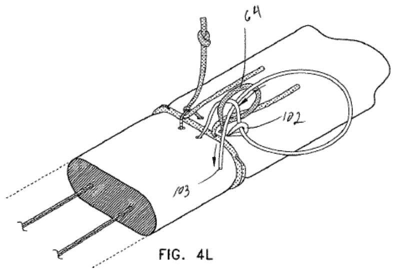

FIG. 4L

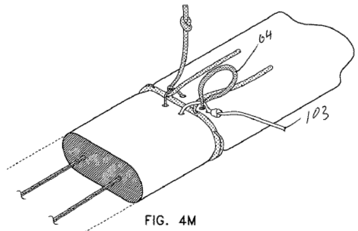

FIG. 4M

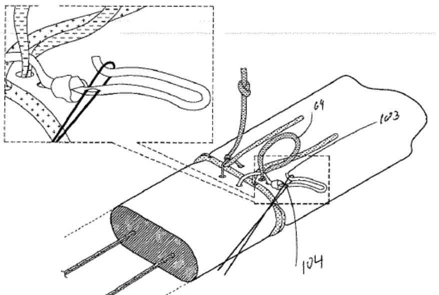

FIG. 4N

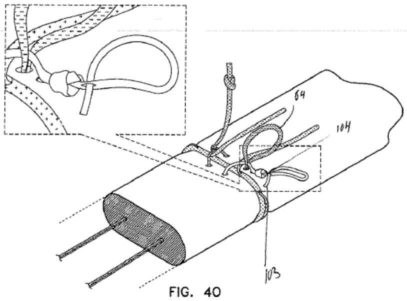

FIG. 4O

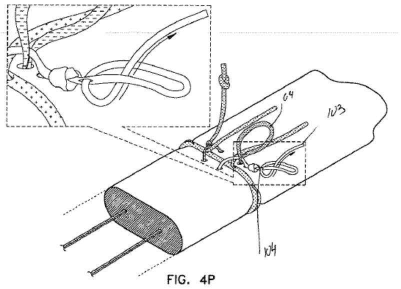

FIG. 4P

Figures 4Q-R illustrate tightening of adjustable self-locking loop 64 of tensioner 50. The same procedure is repeated on the other tendon stump and the fixation system is approximated by pulling free ends 62 of tensioner 50 as shown is Figure 4S.

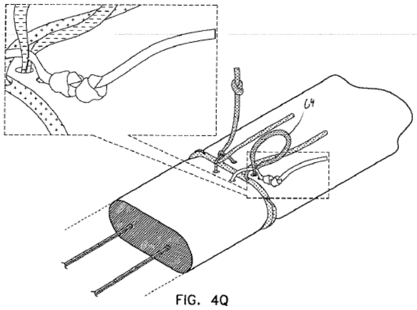

FIG. 4Q

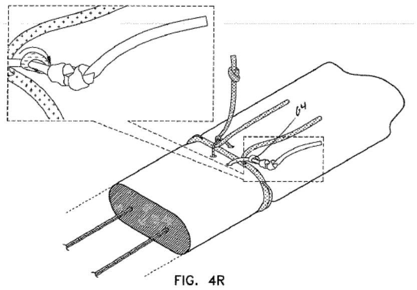

FIG. 4R

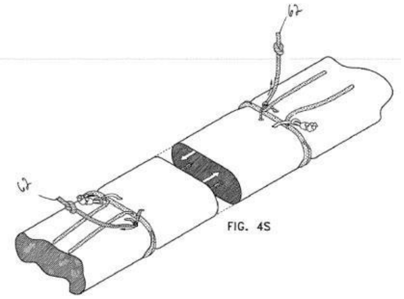

FIG. 4S

-

When the desired approximation is achieved the free ends are cut and the tendon is now repairedas is shown in Figure 4T.

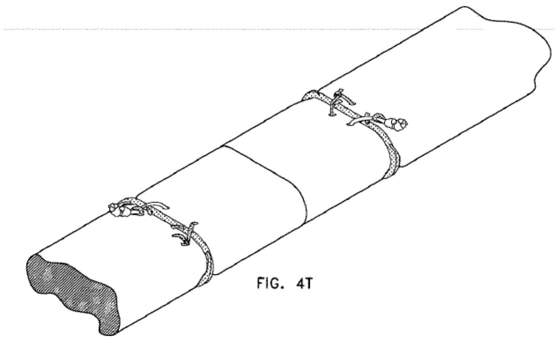

FIG. 4T
